# Prolonged, Atypical, and Fulminant Mpox in a HIV/HBV-Coinfected Patient: A Case Report

**DOI:** 10.7759/cureus.52043

**Published:** 2024-01-10

**Authors:** Felipe de Jesus Perez-Hernandez, Victor Aaron Alvarez-Sanchez, Darwin Torres-Erazo, Monica Ceballos-Perez, Carlos David Franco-Gonzalez

**Affiliations:** 1 Department of Internal Medicine, High Specialty Regional Hospital of the Yucatan Peninsula, Merida, MEX; 2 Infectious Diseases and Epidemiological Surveillance Unit, High Specialty Regional Hospital of the Yucatan Peninsula, Merida, MEX; 3 Department of Clinical Sciences, Universidad Marista de Mérida, Merida, MEX

**Keywords:** monkeypox, hiv, hbv, coinfection, atypical

## Abstract

Mpox (monkeypox) is a zoonotic disease that has been endemic in African countries for decades, with a recent global outbreak in countries around the world. A 39-year-old male with human immunodeficiency virus (HIV)-hepatitis B virus (HBV) coinfection and poor adherence to antiretroviral treatment, who was severely immunocompromised and had a concurrent diagnosis of Mpox, presented to our hospital with disseminated skin lesions (over 350 lesions), perianal ulcers, odynophagia, oral intolerance, diarrhea, and soft-tissue bacterial superinfection of the lower extremities. Laboratory results were consistent with HBV infection, with an absolute CD4 cell count of 40 cells/uL and a positive PCR result for monkeypox virus. An abdominopelvic CT scan showed evidence of severe proctitis and perineal soft-tissue infection. Sixty-five days after a positive monkeypox virus PCR, new lesions in the vesicular stage continued to appear, eventually developing hemodynamic instability and sepsis, resulting in a fatal outcome. Our case highlights the importance of intentionally looking for risk factors such as HIV/HBV coinfection and evaluating immune status (CD4 cell count) in patients with severe Mpox because it could be related to higher mortality.

## Introduction

Mpox (previously monkeypox) is a viral zoonosis with an incubation period that ranges from 5 to 21 days before the onset of symptoms [1,2]. Mpox has been considered endemic in African countries for decades; however, in 2022 different epidemiological studies reported an increase in cases in several countries around the world, and it was declared by the World Health Organization (WHO) as a Public Health Emergency of International Concern in June 2022 [3,4]. Regarding transmission, historically, it has been mainly associated with contact with animal reservoirs such as rodents, and outside of endemic areas, there have been very few person-person cases recorded. In this wave, person-person transmission has been better described as contracting the disease through direct contact with papules, vesicles, respiratory secretions (droplets), and sexual transmission, which has played an important role, associated with a large number of reported cases [3,5]. Proctitis has now been reported in 20% of cases in different series. Fewer cases are recorded in our region and information about clinical manifestations and prognosis is lacking. On the other hand, the information regarding the concomitant infection of Mpox with human immunodeficiency virus (HIV) is still limited and the epidemiological studies published after the recent outbreak identify a large population affected by this coinfection [3]. A case of a Mpox, HIV/hepatitis B virus (HBV) co-infected patient who developed generalized ulcerative injuries, proctitis, and necrotizing fasciitis, ultimately leading to death, in a third-level hospital in Mexico is presented herein in order to highlight the possible synergistic association of a triple viral infection which would imply a greater risk of presenting severe and atypical disease in patients with Mpox.

## Case presentation

**Figure 1:**
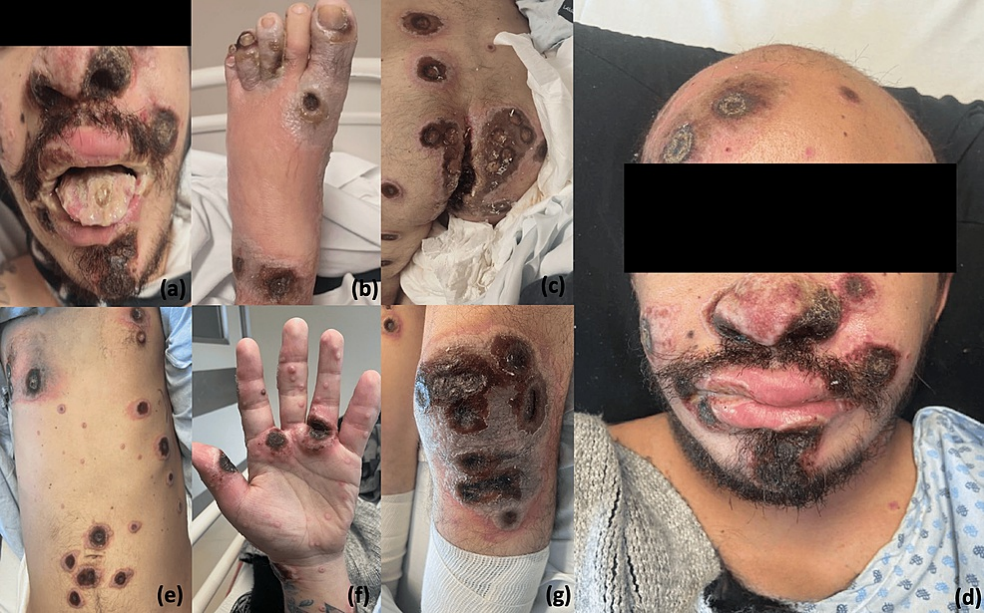
(a) Presence of scars and necrotic ulcers on the face as well as the involvement of the nasal and buccal mucosa with cheilitis. (b) Involvement of the first, third, and fourth toes of the right foot with edema and color changes, as well as the presence of purulent discharge and necrotic ulcers. (c, d, e, f) Disseminated necrotic ulcers in the face, thorax, anterior abdomen, and palms with involvement of the perianal region as well as the rectal mucosa and umbilicated papules around the necrotic ulcers. (g) Erythematous plaque with color changes and edema.

The presented case is of a 39-year-old male with a medical history of HIV infection diagnosed in 2012. He was treated with bictegravir, tenofovir alafenamide, and emtricitabine (single-tablet regimen), but his adherence was poor due to personal issues, mainly poor health-seeking behavior. His HIV status at the time of admission showed virological failure (with a viral load of 100,000 copies/mL), and he was severely immunocompromised (absolute CD4 of 40 cells/uL). He had a history of multiple sexual partners (both same- and opposite-sex partners) and was an active intravenous drug user. Two weeks before admission, the patient presented odynophagia, oral intolerance, diarrhea, and weight loss and said that before the first cutaneous lesions, he had anal intercourse, after which, he started experiencing fever (39.0°C) and a perianal abscess, which was treated with oral antibiotics and drainage. Subsequently, skin lesions appeared, characterized by itchy umbilicated vesicles less than 5mm in diameter, beginning on the face but spreading to the entire body, including the palms and soles, with progression to pustules, scabs, and necrotic ulcers, reaching more than 350 in number by the time of his admission (Figure 1).

**Figure 2:**
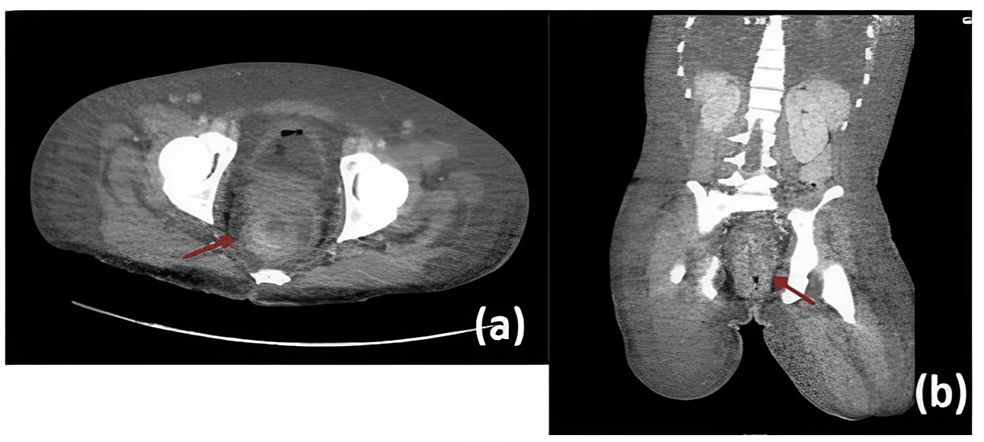
Abdominopelvic CT scan showing inflammatory-looking thickening of the region of the rectum and anal canal (a, b).

**Figure 3:**
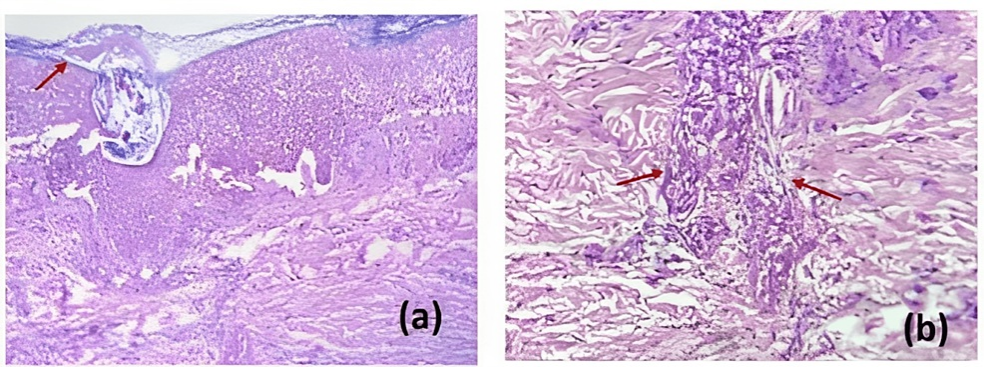
Punch biopsy of umbilicated lesions in the palms with acanthosic epidermis (arrow). (a) Diffuse necrosis of keratinocytes, with full-thickness epithelial involvement, presence of ballooning degeneration in central areas, and intraepidermal bulla formation. (b) Presence of mixed peri adnexal and mild perivascular infiltrate (arrows).

At the time of his hospitalization, we decided to restart his preestablished antiretroviral treatment. Taking into account the immunodeficiency status of the patient and due to the presence of generalized skin lesions and involvement of the lower extremities, severe soft-tissue bacterial superinfection versus primary necrotizing monkeypox was suspected. Therefore, antimicrobial and antifungal treatment was started with cotrimoxazole, piperacillin plus tazobactam, and fluconazole. An abdominopelvic CT scan was conducted, which showed evidence of severe proctitis (Figure 2). New asynchronous lesions continued developing, so skin biopsies were taken (Figure 3). Antibody tests were positive for hepatitis B surface antigen (HBsAg) and hepatitis B e antigen (HBeAg) and negative for cytomegalovirus (CMV), anti-hepatitis C antibody (anti-HCV), VDRL, and FTA-ABS. HBV viral load as well as HBV and HIV resistance genotype to antiretrovirals tests were not available in our hospital. After reviewing the case with a multidisciplinary team (internal medicine, infectious diseases, dermatology, and anorectal surgery), due to a severe and deep infection secondary to monkeypox with the persistence of different stages of new skin lesions 65 days after the initial diagnosis of Mpox by PCR, with the absence of growth in skin cultures, stool, and blood, and without clinical improvement, it was decided to escalate to empirical antibiotic treatment with meropenem, amphotericin B, and cotrimoxazole since the unit did not have antiviral treatment of choice for monkeypox. Despite the above-mentioned treatment, the evolution in his clinical condition was deleterious due to hemodynamic instability, sepsis, and death. New asynchronous Mpox vesicular skin lesions continued to appear until the time of his death. It is important to note that during this patient´s hospitalization and clinical development of Mpox, the specific antiviral treatment was still in phase III of the clinical trials in foreign countries but was not available or approved for use in Mexico.

## Discussion

The Americas have been the most affected region in the recent Mpox outbreak, accounting for 67.6% of cases and 71% of deaths worldwide (WHO as of May 9th, 2022). Epidemiological reports on the current Mpox outbreak identify men (~97%), with a median age of ~34 years, and a high proportion of them living with HIV (36-67%), as the most affected group [5,6]. Despite this, mortality is low (< 0.1%), and extensive (> 100 lesions) and severe presentations (confluent lesions, sepsis, or the involvement of risky anatomic areas such as the eyes, anus, etcetera) are rare and have been associated with immunocompromised individuals [5,6]. In a CDC weekly report, cases of 57 hospitalized patients with severe Mpox were reviewed, where there were 12 deaths (21%), and it was noted that 82.5% were persons living with HIV and 72.1% had a CD4 count < 50 cells/mm^3^ (97% < 200). This contrasts with the mortality rates reported in studies of patients with CD4 counts averaging 462 cells/mm^3^ (0.17% mortality), 510 cells/mm^3^ (0.0% mortality), or 664 cells/mm^3^ (0.0% mortality) [6-9].

Our patient, who unfortunately died, had an extensive presentation of >350 confluent lesions and severe rectal complications and was probably experiencing virologic failure despite antiretroviral treatment and severely compromised immunity (CD4 of 40 cells/uL). In the response to Mpox infection, effector CD4 T cells could play a central role in the memory and differentiation of B lymphocytes to antibody-producing cells. Likewise, probably there is an immune cellular response to Mpox infection characterized by the rapid expansion of activated effector CD4 and CD8 cells, which generate a Th1 immune response (with the production of cytokines such as IFN-γ, IL-1β, IL-6, IL-8, TNF, and MCP-1). This response is observed in HIV patients with CD4 > 350 cells/mm^3^; however, there are no data regarding patients with CD4 < 350 cells/mm^3^ [10].

In any case, HIV/HBV coinfection has been shown to be associated with increased risk of advanced HIV disease and lower CD4 count at diagnosis, as well as poor immune response and significantly decreased CD4 count recovery after antiretroviral therapy [11-13].

Therefore, it is possible that appropriate humoral and cellular responses against Mpox were hampered due to the low CD4 levels secondary to virological and immune failure and exacerbated by HIV/HBV coinfection, leading to active replication of Mpox (by the presence of new vesicular phase lesions even after four weeks of onset), and ultimately to death; although this was not completely attributable to Mpox as a cause, the latter was probably a highly contributing factor [14].

To our knowledge, severe, extensive, and fatal Mpox infection, with the risk factors of HIV/HBV coinfection and immune failure despite antiretroviral treatment, has not been reported, which highlights the importance of intentionally searching for such coinfections.

## Conclusions

In the face of severe Mpox, it is important to intentionally search for coinfections such as HIV and HBV, as well as to conduct evaluation of immune status (CD4 cell count), since a deteriorated immune status (CD4 <200cells/mm^3^) seems to be related to higher mortality; however, large epidemiological studies are required to corroborate this association.
